# Wind Power and NIMBYism in Norway: Public Attitudes and Local Resistance

**DOI:** 10.1007/s00267-025-02121-5

**Published:** 2025-01-31

**Authors:** Krange Olve, Figari Helene, Kaltenborn Bjørn

**Affiliations:** 1 Norwegian Institute for Nature Research (NINA), Oslo, Norway; 2 Norwegian Institute for Nature Research (NINA), Lillehammer, Norway

**Keywords:** NIMBY, Wind power, Attitudes, Local resistance vs. general values, Norway

## Abstract

This study explores the impact of NIMBY (Not In My Backyard) sentiments on local resistance to wind power developments in Norway. With a reopening of concession processing for new onshore wind power projects in 2022, a significant number of applications await evaluation, provoking substantial local opposition. This research assesses the prevalence and impact of NIMBY attitudes among the Norwegian public and to critically examine the theoretical validity and practical utility of the NIMBY concept. Using data from an online survey, which yielded 1220 complete responses, we analyzed general attitudes toward wind power in Norway and specific attitudes toward local wind power installations. While 37% of respondents support wind power construction in Norway, only 27% favor it near their homes. Based on the relationship between these two attitudes, we identified the proportion of NIMBYs in the data using two approaches. A strict definition requires individuals to support wind power in Norway but oppose its presence in the natural areas near their own homes. A less strict definition also includes those who expressed a neutral stance toward wind power in Norway among the NIMBYs. In both cases, a relatively small segment of respondents exhibit classic NIMBY characteristics, i.e., support (or claim neutrality) to wind power in general but opposing it locally. Further analysis reveals that direct experience with wind power installations is associated with increased acceptance rather than opposition, challenging the NIMBY narrative. Our study argues that labeling local resistance as NIMBYism oversimplifies the issue and ignores other significant factors like environmental identity, place attachment, and broader environmental attitudes. Hence, the study suggests that blaming wind power opponents as “Nimbys” often is misplaced and unjust.

## Introduction

During spring 2022, and following a break exceeding three years, the Norwegian Water Resources and Energy Directorate (NVE) recommenced the concession processing of plans for new onshore wind power developments. At present, nearly 30 applications await evaluation within the directorate, with additional proposals in various stages of development in the pipeline. Like in most other countries, a dominant political narrative states that Norway needs an increased supply of environmentally sustainable energy, commonly referred to as “green energy”, to fulfill its mandated reduction in CO_2_ emissions. Nevertheless, new initiatives for wind power plants consistently provoke substantial local opposition. Could the protests we are witnessing be indicative of the so-called NIMBY effect?

In a widely cited article, (Dear 1992, p. 288) explains NIMBY as “the motivation of residents who want to protect their turf”, acknowledging that these “noxious” facilities are necessary, but not near their homes, hence the term “not in my back yard”. In other words, the term points to attitudes on two levels, locally and more generally (for example, nationally). Hence, the NIMBY acronym suggests that people may be positive to something at a theoretical level (such as the presence of wind power plants in Norway) but simultaneously reject it when it appears or is placed in locations that directly affect them (for example, wind power plants in the vicinity of their own homes). Hence, NIMBY is about accepting something on a rather abstract or theoretical level but opposing it in practice when placed in one’s own neighborhood. Therefore, attributing NIMBYism as an explanation for people’s attitudes or labeling individuals as “Nimbys” often conveys a notion of self-interest, selfishness and hypocrisy (Wolsink 2000), and has been described as a strategy for stigmatizing and delegitimizing local resistance in the context of wind power development (Haikola 2023).

In Norway, wind power development continues to fuel a heated discussion with accusations of NIMBYism frequently present in public debate as a primary explanation for local resistance to specific wind power development plans (Gammelsæter and Lorange 2019). These claims not only imply that NIMBY sentiments are a critical factor in understanding resistance but position them, and hence selfish hypocrisy, as the fundamental reason behind the existence of opposition in the first place (Batel and Devine-Wright 2020). At present, there is little evidence to suggest that the conflicts will diminish in the near future. Moreover, we lack sufficient scientific understanding to definitively identify which geographical, cultural, social, or psychological factors are the most critical in this complex issue.

Indeed, research evidence scarcely supports the notion that local opposition to wind power stems from selfishness and hypocrisy. Instead, the social acceptance of renewable energy emerges as a complex attitude expression. Scholars have argued that the NIMBY concept may be overly simplistic and inadequate for explaining attitudes, concerns, motivations, and beliefs that lead to both negative feelings and expressions (Devine-Wright 2009; Rand and Hoen 2017; Vasstrøm and Lysgård 2021), and questioned the credibility of NIMBYism as a reliable explanation for opposition toward wind power (Petrova 2016).

Many of these arguments have been articulated within the realm of qualitative research, which often delves deeply into the socio-cultural contexts and underlying motivations of wind power resistance (Batel and Devine-Wright 2020). Meanwhile, quantitative survey research on the relationship between proximity to wind power projects and increased opposition has produced confounding results (Rand and Hoen 2017), with some studies finding a significant positive correlation (e.g., Swofford and Slattery 2010), while others report no clear pattern or even opposite findings, where closer proximity correlates with greater acceptance (Hoen et al. 2019; e.g., Warren et al. 2005).

Carley et al. (2020) conducted a systematic review revealing that surveys on NIMBYism in energy projects primarily focus on wind energy, reflecting the common tendency to portray opposition to wind power as NIMBY. They identified three survey approaches to the study of NIMBYism in energy infrastructure. The *over-sampling approach* concentrates on populations residing near existing or proposed energy projects, aiming to assess their level of support in comparison to those living at a greater distance. The *distance approach* evaluates either the physical or perceived proximity between individuals and energy projects to determine if closeness affects their support or opposition. The *dependent variable approach* involves directly questioning participants about their willingness to support energy projects located near their homes, thus using hypothetical scenarios to assess the influence of proximity on their acceptance.

Additionally, Carley et al. (2020) identified significant shortcomings and challenges in the study of NIMBYism within energy infrastructure research, emphasizing the inconsistent application and measurement of NIMBY sentiment as a primary concern. They note that the term “NIMBY” is broadly used to signify local opposition, but its precise meaning and how it is operationalized varies widely across studies, often neglecting the classical definition that encompasses supporting a technology in principle while opposing its local implementation. Furthermore, the authors point out the diverse and non-standardized methods employed to measure geographic proximity to energy projects and select study populations. Such variability complicates efforts to compare results across studies or generalize findings, particularly when studies do not include comparison groups that could illuminate how proximity influences support or opposition. In short, the authors call for more standardized methodologies for defining NIMBYism and measuring proximity.

The research presented here adopts the “dependent variable approach”, which involves directly asking respondents about their support for energy projects in areas they perceive as near their homes. While Carley et al. emphasizes the importance of combining the approaches mentioned above and advocate for greater integration across studies, they also recognize the dependent variable approach as an especially useful tool for elucidating the complex relationship between general support for an energy technology and specific opposition to its local implementation. Our study furthermore adheres to the classical dual definition of NIMBYism.

However, the study does not employ a standardized method for measuring geographical proximity. Indeed, and as pointed out by Carley et al. (2020), one limitation of the dependent variable approach is the lack of variation in the distances considered, which restricts the ability to explore how support changes across different proximities. Furthermore, the absence of a standardized distance metric in this approach limits comparability across studies, reducing our possibility to assess the importance of proximity.

In the following sections we present the design and results from the Norwegian study, discuss our methodological choices, and argue why standardized measures of geographical proximity might not always serve to advance the understanding of NIMBYism.

### Aim

Our objective is to assess the impact of NIMBY sentiments on the resistance to wind power in Norway. We ask: What percentage of individuals who express a generally positive attitude toward plans for wind power in Norway also express negativity when it comes to having wind turbines in the natural areas close to where they live? Based on this we identify respondents that may be identified as Nimbys and ask if the likelihood of falling into the NIMBY category is greater for those who live in areas where there are plans for or in areas where wind power plants already exist? This approach further provides a basis for critically assessing the theoretical validity and practical utility of the NIMBY concept in a Norwegian context.

## Material and Methods

We gathered data through an online questionnaire conducted from June to July 2021, utilizing the online survey platform and panel Qualtrics (qualtrics.com). The sample was designed to represent the Norwegian public on a national scale, employing stratification based on age, gender, and region. Our survey yielded 1220 fully completed responses, resulting in a response rate of 83%. Among the respondents, 48% identified as female, and 51% as male. The average age was 46, slightly above the official average for 2023 (Statistics Norway n.d.-a). The majority reported an annual household income between 400,000 to 600,000 NOK. In 2022, the median household income for all Norwegian households was NOK 592,400 (Statistics Norway n.d.-b). Since the sample was stratified to represent the general public across the nation, the resolution of the data is not sufficiently high to permit correlation with specific geographic locations.

To capture the overall attitude toward wind power, we asked whether respondents liked or disliked the construction of wind power in Norway in general: “What do you think about building wind power plants in Norwegian nature?” To be able to address the NIMBY issue, we tapped into what respondents thought about wind power developments close to where they live, we asked: What do you think about building wind power plants in the natural areas near where you live? In Norway, wind power developments are invariably located in what may be termed nature or natural areas, which is why we used the term *nature* in the questionnaire. The strategy enables us to assess respondents’ opinions on wind power plants that they perceive as being close to where they live. Responses to both questions were recorded on a 5-point Likert scale, ranging from 1 (like very much) to 5 (dislike strongly). We used a combination of these two questions to assess the percentage of “Nimbys” in our sample. Moreover, NIMBY is primarily a term coined to describe resistance among people affected by developments that are generally considered to be desirable. To capture this aspect respondents were asked: “Are there already wind power plants in your local area, or are there any plans for new developments?” Respondents could choose between three possible responses: “Yes”, “No” or “Don’t know”.

Carley et al. (2020) observed significant heterogeneity in how empirical research on NIMBY is designed, particularly in how researchers have approached and measured geographical proximity. In our approach to this question, we have implicitly distanced ourselves from methods that involve measuring the actual physical distance between individuals’ homes and an existing or planned wind farm. There are several reasons for this.

One reason is that the importance of distance, as measured in meters or kilometers, may be of minor relevance to people’s perceptions and concrete experiences. Variations in landscape and topography can entail that a distance of several kilometers may have a greater impact on people’s quality of life than facilities located closer by. Much of the wind power development in Norway has occurred along the coast, a landscape characterized by bare mountains and rocks, sea, and fjords. A wind farm “behind the hill” versus “right in your face on the other side of the fjord” can for instance result in vastly different experiences.

This suggests that people’s subjective experience of “natural areas near where you live” aligns better with what we are aiming to capture. A non-contextual or standardized approach to assessing distance, such as applying a quantitative measure, can also be misused by wind power advocates to label opponents as selfish, obstructive, or insensitive to the needs of greater society. Such an approach does not account for the lived experience of having wind power installations in one’s vicinity. Therefore, there is a moral aspect to consider, and we caution against using physical distance rather than the subjective experience of presence as a valid indicator of impact or influence.

## Results

Table 1 shows the distribution of the overall view of the public toward wind power in Norway, both as developments in Norway in general and in the vicinity of the respondents’ homes. Thirty-seven percent claimed they like or like very much wind power construction in Norway in general, compared to only 27% that liked the idea of having them near their own homes. On the other end of the scale, we observed that 34% stated that they dislike or dislike wind power strongly in general and that 44% expressed such skepticism (dislike or dislike strongly) about wind power close to where they live. At both ends of the scale, the difference was 10% points. Almost one-third (29%) seemed to be indifferent to both questions.

**Table 1:** Attitudes toward wind power in Norway (percent)

	What do you think about building wind power plants in Norwegian nature?		What do you think about building wind power plants in the natural areas near where you live?	
	%	*N*	%	*N*
Like very much	15	179	10	126
Like	22	263	17	212
Neutral	29	355	29	340
Dislike	17	207	20	246
Dislike strongly	17	212	24	292

These distributions confirm that Norwegians hold divergent opinions toward onshore wind power. This is evident not only at the national level but also in the respondents’ reactions to the idea of having such facilities in what they perceive as in the proximity to their own homes. However, it is equally evident that general plans for wind power establishment gained more support and encountered less skepticism than the idea of having such developments near one’s residence.

In the subsequent analyses, we examined the proportions of the observed difference in responses to the two attitude questions that can be explained by the NIMBY effect (Table 2). We applied two different definitions of NIMBY:

- Strict definition: respondents who liked or liked very much wind power in Norway while simultaneously disliking or strongly disliking having them in nature close to home.
- Less strict definition: same as above, but with the addition that those who stated that they were neutral to the presence of wind power in Norway were also included in the analysis.

**Table 2:** Percentage of Nimbys by two definitions

	Qualified	Potential	Percentage of “Nimbys”
Strict definition	32	442	7
Less strict definition	132	797	17

**Fig. 1:**
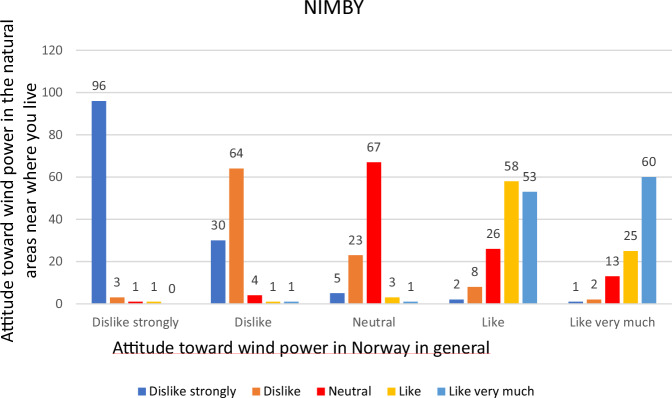
Attitudes to wind power in natural areas close to where the respondents live by attitudes to wind power in Norway general. (percent) Pearson Chi-Square: 2062.01, *p* < 0.001

Given the complexity of the NIMBY concept and the lack of clear consensus in the literature on antecedents, structure, and definitions, we decided to include a less strict definition as well since we suspect that people without a clear stance on wind power may well oppose it if they are exposed to it in their immediate surroundings. In other words, including two related definitions of NIMBY might help clarify the substantive nature of the concept (Fig. 1).

The figure illustrates the relationship between respondents’ answers to the question about liking wind power plants in Norway in general and the question about having them in the natural surroundings where one lives. For example, we can observe that 96% of those who strongly disliked the presence of wind power in Norway also indicated a strong dislike for the idea of having them in their own vicinity. Similarly, 60% of those who liked very much the presence of wind power in Norway also expressed that they would “like very much” having them in their surroundings close to where they live. In the same group, only 1% indicated a strong dislike for having them in their nature. Not surprisingly, there is a high correlation between the responses to the two questions, especially among those who disliked the idea of wind power existing in Norway. This may seem obvious, but the focus here is on what happens at the other end of the scale, among those who liked the presence of wind power plants in Norway. This is where we find those who may be labeled “Nimbys”.

The term NIMBY can be operationalized in various ways. An initial starting point could be that so-called “Nimbys” are those who like or like very much having wind power plants in Norway while simultaneously disliking or strongly disliking having them in the natural surroundings where they live (strict definition). With this operationalization, there are only 32 respondents in our data who qualify as “Nimbys”. They constitute less than 3% of the sample (*n* = 1220). However, it is simply illogical to include those who, by this definition, cannot have NIMBY perceptions—those who dislike the presence of wind power in Norway. 442 respondents indicated that they either like or like very much the presence of wind power plants in Norway. Based on this segment of the sample, the 32 respondents would make up 7% (*n* = 442).

One could argue that the definition above is overly strict and that even those who take a neutral position on wind power plants in Norway and simultaneously dislike having them in their local surroundings should be included in the definition. With these less strict criteria, 132 respondents meet the NIMBY requirements. This constitutes 17% of the subsample consisting of those who answered, “like very much”, “like” or “neutral” (*n* = 797) on the question about wind turbines in Norway. With the less strict definition, there is a larger proportion that can be characterized as Nimbys, but still less than one in five.

**Fig. 2:**
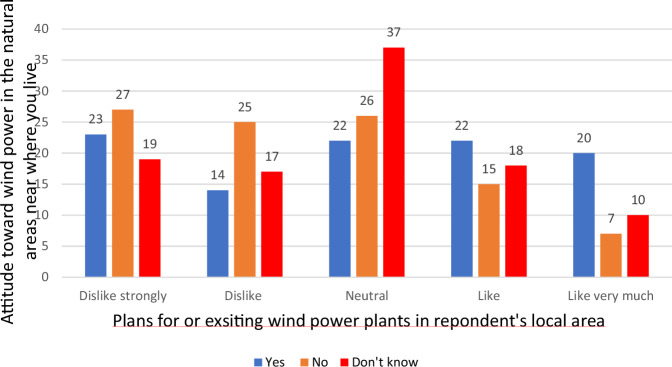
Attitudes toward wind power by plans for or already existing wind power near respondent’s local area (percent) Pearson Chi-Square: 70.85, *p* < 0.001

In the study of opposition to energy technologies NIMBY has predominantly been employed to explain—or, one might argue, to rationalize—local opposition, particularly to wind power when plans for development are on the table (see e.g., Devine-Wright 2009). Both attitudinal measures examined in the preceding analyses are based on respondents’ abstract ideas and conceptual considerations rather than practical or lived realities. Neither the question regarding the respondents’ general stance on wind power in Norway nor the prospect of wind power plants presence close to where they live ask for their concrete experiences. Favoring the thought of its existence in the surroundings of one’s own home does not equate to its factual presence. Figure 2 shows the distribution of respondents classified as Nimbys in relation to the question whether wind power is established or planned in the respondents’ own vicinity. Even if NIMBYism is not very widespread it might be more prevalent among respondents that have concrete experiences with wind power plants. To achieve sufficient statistical impact, we used the less strict definition.

Before proceeding, we take a brief detour to examine the relationship between attitudes toward wind power in one’s local surroundings and the respondents’ responses to the question of whether wind power or any plans already exist for the area where they live.

The general distribution for the question “What do you think about the construction of wind power close to where you live?” indicated that “dislike” or “strongly dislike” responses are more prevalent than “like” or “like very much.” The same is not true for respondents who stated that wind power or plans for wind power development exist in their vicinity. In this group, 42% of respondents were positive, and only 37% were negative about wind power in their local area. For the group that answered “no” to the same question, negative attitudes are significantly more widespread. Among these respondents, only 22% were positive, while a substantial 52% expressed negative attitudes. In other words, concrete experiences with wind power, as measured here, do not seem to predicate negative attitudes toward wind power development. Quite the opposite, it appears. NIMBYism can, of course, still be highly prevalent locally. However, this requires that NIMBY attitudes are quite infrequent among respondents living in areas where wind power does not currently exist and where no plans for such development are in place.

**Fig. 3:**
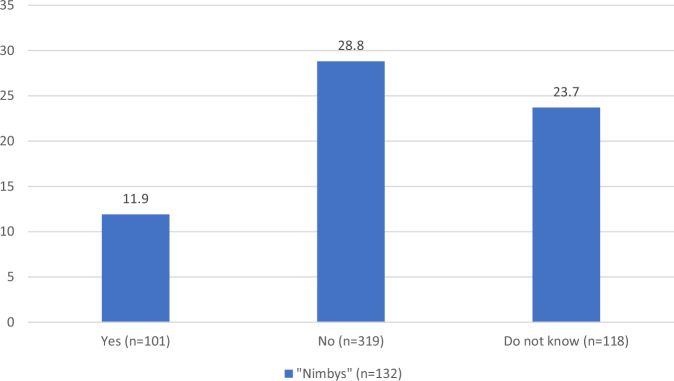
Percentage of Nimbys by plans for or already existing wind power near respondent’s local area. Sample consists only of those who expressed negative sentiments toward wind power near residency (percent, *n* = 538)

Obviously, the NIMBY category exclusively includes individuals who hold negative sentiments regarding the prospect of wind power constructions close to where they live. Figure 3 depicts the outcomes of an analysis wherein the cohort is refined to solely comprise respondents indicating disapproval, either strong or moderate, toward the installation of wind power in their immediate vicinity. The question at hand is whether the proportion of Nimbys is greater among those who report that wind power construction is already established or that plans exist for such development in their local area.

All the respondents included in this analysis expressed a negative view toward wind power in the areas near their residences. Among those who reported no existing facilities or plans for new wind power installations in their local surroundings, nearly 30% can be categorized as Nimbys. Conversely, this is true for only 12% among those indicating the presence of existing facilities or plans for such installations. In light of the findings displayed in Table 2, this is not particularly surprising; however, the numbers do not suggest that NIMBYism constitutes a substantial portion of local opposition to wind power in Norway. NIMBYism, as measured here, is simply more prevalent among respondents who do not have direct experience with wind power in their local area.

## Discussion

We had two main objectives with this study: first to identify the portion of the Norwegian public who could be said to express NIMBY attitudes in relation to wind power development and how NIMBYism is related to living in areas with existing or planned wind power plants. Second, to critically assess the NIMBY concept and its theoretical validity and the practical usefulness in an applied perspective in the controversies around renewable energy development.

In studies of controversial topics with broad societal relevance, there is always a danger of social desirability biases. A problem that may occur when we ask questions the way we do in this study is that it may invoke the so-called Hawthorne effect (McCambridge et al. 2014). This phenomenon occurs when respondents tailor their answers to present themselves in a more favorable light, potentially leading to an underestimation of the prevalence of NIMBYism.

However, since the sample is drawn from a panel of individuals experienced in responding to questionnaires, respondents in this study were well aware that all data would be anonymized, allowing them to answer confidently without fear of identification. This suggests that they likely trusted the data-handling process enough to provide honest responses. Additionally, our findings are by no means counterintuitive. The notion that NIMBY and NIMBYism explain little of the local resistance to wind farm developments is a rather common finding in research within this domain (Figari et al. 2024; Niskanen et al. 2024a; Petrova 2013; Rand and Hoen 2017).

We also recognize that NIMBY, as defined and operationalized here, can be a completely legitimate stance (Feldman and Turner 2014; Niskanen et al. 2024b; van der Horst 2007) For instance, it is likely that most people agree that a modern society needs airports, roads and waste management facilities. One would probably not label individuals who oppose having an airport in their residential area as Nimbys, calling them selfish or lacking concern for greater society. What then is the basis for characterizing local opposition to wind power as NIMBY? Based on our findings and interpretations, we portend that wind power can be merely a minor inconvenience rather than a significant issue. The crux of the issue is whether wind power installations are a substantial burden on nature and human communities or not. In addition, there is a substantial body of research indicating that NIMBY explains little of the local resistance (Batel and Devine-Wright 2020; Burningham et al. 2015; Jarvis 2024; Schwenkenbecher 2017). On the contrary, other phenomena, such as perceptions of local environmental quality and trust in energy companies, have been shown to have predictive value or explanatory power (e.g., Konisky et al. 2020). Perhaps a better way of thinking about NIMBY is that this attitudinal expression is a symptom of discontent with a series of conditions linked to planning, design, decision-making, altering of the meaning of landscapes, and/or disagreement with energy policies.

As touched upon in the Introduction, NIMBY as a concept of how people relate to a complex issue such as wind power may turn out to have little predictive value since it likely embraces “apples and oranges” in the sense that it seeks to form a collective expression of fundamental attitudes toward nature, sense of place, personal experiences with environmental impacts, beliefs about sustainability and appropriate technology. This could mean that NIMBY as a concept covers up so many different emotional, cognitive, and cultural components that are potentially contradictory, that it precludes identifying particular and more important patterns in public attitudes, for example, place attachment (Dugstad et al. 2022).

Another aspect that is worth further attention is the question of habituation to change. Interest in the temporal dynamics of attitudes and exposure to wind power turbines is increasing within the field of wind power research (le Maitre et al. 2024; Wilson and Dyke 2016; Windemer 2023). Some studies indicate that firsthand experiences with wind power generally lead to greater favorability toward it after project completion (Wilson and Dyke 2016). However, other studies provide less conclusive results, creating uncertainty about whether living near wind power turbines reduces or heightens resistance over time (Zerrahn 2017).

On a more general level, insights from other research areas on environmental impacts, risk perception, and adaptive behavior can be valuable for improving our understanding of questions related to exposure and habituation. Studies from other fields suggest that people who live close to potential danger or rapidly changing environmental conditions may sometimes cope better with change and in some cases express lower levels of fear or distress. However, the idea that people can always adapt, or that one impacted area can be substituted by providing new opportunities somewhere else, is clearly overly simplistic. Assuming that individuals should accept inconveniences or even major detriments to quality of life on behalf of society is a morally questionable argument since economic gain is pitted against non-economic values. Also, in many cases, people will not actually have the freedom to choose alternatives or the mental, physical, or economic capacity to absorb changes in a satisfactory way. This is especially evident in the presence of wildlife that can cause a threat to human safety and people who live in areas that are more prone to natural hazards. Researchers have ventured different explanations for the seemingly contradictory situation that proximity to potential danger or impacts of rapid change elicits reduced distress and indirectly increased acceptance. Prominent among these is the role of place attachment, i.e., strong connections to one’s immediate environment, and that being exposed to potential problems where little or nothing of real significance actually happens can act as a form of exposure therapy and reduce phobia.

These psychological mechanisms may also have relevance for wind power development in the sense that many people adapt to changes in their surroundings as time goes on, and when they gain concrete experiences, rather than relying on abstract images or ideas about what the future may look like. We portend that the combination of the “fuzziness” and lack of precision in the NIMBY concept, and the real-life experiences of people who live in close proximity to wind parks, maybe at least part of the explanation for the low percentages of NIMBYs identified in this study.

It is worth noting that the data used in this study were collected before the Russian invasion of Ukraine. In 2018, a solid majority of Norwegians (65%) thought that Norway should increase wind power production (Aasen et al. 2022). Four years later, in 2021, this proportion was nearly halved to 33%. However, recent data suggests a reversal of this trend, with support for new wind power installations increasing to 39% in 2022 and 42% in 2023 (CICERO n.d.) The war in Ukraine has led to significantly increased demand for energy produced in Europe. This geopolitical situation may have contributed to the changing sentiment in Norway, suggesting that we might be underestimating the current level of general support for onshore wind power. Additionally, we have no information on how acceptance of having wind power plants near one’s own residence may have changed, but if acceptance of such nearby installations has remained stable, it would imply that the proportion of “Nimbys” is even lower than our estimates suggest. Moreover, it may also be considered a limitation to the study design that we ask if respondents favor the thought of wind power installations surroundings of one’s own home. The impact of the actual presence of such installations on attitudes falls beyond the scope of what the data can reveal. It is, however, worth noticing that resistance to wind power projects tends to decrease for respondents who live near a wind power plant or in an area where there are plans for such installations.

Outside the realm of science, NIMBY is sometimes used as a convenient explanation for opposition to various forms of infrastructure development. However, the term is frequently applied too simplistically, ignoring that it encompasses attitudes on both local and general levels. This oversimplification can have undesirable social and political consequences, as interests behind various developments may claim undue legitimacy. We argue that NIMBY glosses over the complexity of attitudes to such an extent that it becomes of limited usefulness in many cases. Based on the same dataset that we use in this study, we have previously observed that Norwegians’ attitudes toward wind power are closely associated with environmental identity (Keller et al. under review) and broader environmental attitudes, such as perceptions of energy policy, social and ecological consequences of siting, place attachment, and more fundamental worldviews such as eco-modernism versus de-growth (Kaltenborn et al. 2023).

## Conclusion

Several studies on NIMBY in the context of wind power development have suggested that local resistance represents more than a desire or demand to avoid the burdens of something perceived as positive at a more general level (Devine-Wright 2009; Petrova 2016; Rand and Hoen 2017; Vasstrøm and Lysgård 2021). In consistence with this, our findings indicate (1) that NIMBYism is generally infrequent, and (2) that local opposition to wind power only to a small degree is associated with NIMBYism. Based on the presented findings, we conclude that accusations of NIMBYism, often directed toward local anti-wind power groups, are generally misplaced, unjustified, and potentially with negative social consequences. While developers, contractors, and their allies may find it useful to portray their adversaries as self-interested, egocentric, and hypocritical, such characterizations lack substantive support from the numerical data presented in this study. This moral aspect of the wind power debate still requires more thorough scrutiny and attention. Even though it has become mainstream within the social sciences to abandon the NIMBY explanation for local resistance to land-based wind power developments, it persists among those promoting such projects. Decisions on infrastructure that significantly impact both nature and society should not be based on false assumptions about a high prevalence of NIMBY attitudes.

## Data Availability

No datasets were generated or analysed during the current study.

## Appendix

**List of the questions presented to the respondents**

Analysis in this paper is based on 1220 Norwegian respondents’ answers to the three questions presented below. The original Norwegian questions are quoted together with our translation.

Hva syns du om at vi bygger vindkraftanlegg i norsk natur?

Translates to:

What do you think about building wind power plants in Norwegian nature?

Scale: (1) Like very much, (2) Like, (3) Neutral, (4) Dislike, (5) Dislike a lot

Hva syns du om å bygge vindkraftanlegg i naturen nær stedet du selv bor?

Translates to:

What do you think about building wind power plants in the natural areas near where you live?

Scale: (1) Like very much, (2) Like, (3) Neutral, (4) Dislike, (5) Dislike a lot

Er det alt etablert eller finnes det planer for etablering av vindkraftanlegg i ditt nærområde?

Translates to:

“Are there already wind power plants in your local area, or are there any plans for new developments?”

Values: (1) Yes, (2) No, (3) Don’t know
